# Inhibition of Thioredoxin-Reductase by Auranofin as a Pro-Oxidant Anticancer Strategy for Glioblastoma: In Vitro and In Vivo Studies

**DOI:** 10.3390/ijms26052084

**Published:** 2025-02-27

**Authors:** Nelly Chmelyuk, Maria Kordyukova, Maria Sorokina, Semyon Sinyavskiy, Valeriya Meshcheryakova, Vsevolod Belousov, Tatiana Abakumova

**Affiliations:** 1 Department of Synthetic Neurotechnologies, Pirogov Russian National Research Medical University, 117997 Moscow, Russia; 2 Laboratory of Biomedical nanomaterials, National Research Technological University “MISIS”, Leninskiy Prospekt 4, 119049 Moscow, Russia; 3 Neurotechnology Laboratory, Federal Center of Brain Research and Neurotechnologies, Federal Medical Biological Agency, 117513 Moscow, Russia

**Keywords:** auranofin, glioblastoma, thioredoxin reductase, redox system, glioma stem cells

## Abstract

Reactive oxygen species (ROS) play a key role in cancer progression and antitumor therapy. Glioblastoma is a highly heterogeneous tumor with different cell populations exhibiting various redox statuses. Elevated ROS levels in cancer cells promote tumor growth and simultaneously make them more sensitive to anticancer drugs, but further elevation leads to cell death and apoptosis. Meanwhile, various subsets of tumor cells, such a glioblastoma stem cells (GSC) or the cells in tumor microenvironment (TME), demonstrate adaptive mechanisms to excessive ROS production by developing effective antioxidant systems such as glutathione- and thioredoxin-dependent. GSCs demonstrate higher chemoresistance and lower ROS levels than other glioma cells, while TME cells create a pro-oxidative environment and have immunosuppressive effects. Both subpopulations have become an attractive target for developing therapies. Increased expression of thioredoxin reductase (TrxR) is often associated with tumor progression and poor patient survival. Various TrxR inhibitors have been investigated as potential anticancer therapies, including nitrosoureas, flavonoids and metallic complexes. Gold derivatives are irreversible inhibitors of TrxR. Among them, auranofin (AF), a selective TrxR inhibitor, has proven its effectiveness as a drug for the treatment of rheumatoid arthritis and its efficacy as an anticancer agent has been demonstrated in preclinical studies in vitro and in vivo. However, further clinical application of AF could be challenging due to the low solubility and insufficient delivery to glioblastoma. Different delivery strategies for hydrophobic drugs could be used to increase the concentration of AF in the brain. Combining different therapeutic approaches that affect the redox status of various glioma cell populations could become a new strategy for treating brain tumor diseases.

## 1. Redox Balance in Glioblastoma and the Role of ROS in Glioma Stem Cells and Tumor Microenvironment

Glioma is the most common primary malignant brain tumor in adults. This condition generally has a poor prognosis, especially for the most aggressive form of glioma, glioblastoma (GBM), where the median survival time after diagnosis is approximately 14 months despite standard modern treatments, including surgical tumor removal followed by radiation and chemotherapy [1]. Therefore, the development of new therapeutic strategies to treat this disease is urgently needed.

One of the key reasons for chemoresistance in GBM cells is their insusceptibility to oxidative stress caused by elevation of cellular ROS level. ROS participate in mediating the cytotoxic effects of various treatments [2]. For example, the main chemotherapeutic agent for GBM, temozolomide (TMZ), has been shown to induce ROS production in addition to its alkylating action [3,4,5]. Furthermore, rapid and repeated increases in ROS caused by TMZ can activate autophagy [5], apoptosis, and cell death [6].

The primary ROS in cells include superoxide radicals, hydrogen peroxide, and hydroxyl radicals. Cellular antioxidant enzymes such as superoxide dismutase, catalase, thioredoxin reductase, glutathione reductase, peroxiredoxins, and glutathione peroxidases, as well as cofactors such as nicotinamide adenine dinucleotide (NADH) and nicotinamide adenine dinucleotide phosphate (NADPH), regulate physiological ROS concentrations [7]. Notably, many cellular antioxidant systems are functionally interconnected and often compensate for one another. The balance between antioxidant system activity and intracellular ROS concentrations is referred to as the redox status of the cell [8].

Cancer cells contain higher levels of ROS than normal cells. Sources of increased ROS production in tumor cells have been attributed to oncogene- and damage-stimulated production of O_2_ by mitochondria, associated with altered assembly of the electron transport chain, hypoxia; O_2_ by NADPH oxidases (NOX); H_2_O_2_ by 5-lipoxygenase; and H_2_O_2_ within the endoplasmic reticulum [9]. A mild increase in ROS stimulates the proliferation of cancer cells by inhibition of PTEN [10] and MAPK phosphatases [11]. To avoid cell death due to such an increased ROS level, cancer cells activate antioxidant defense systems. Since the toxic threshold of ROS levels in cancer cells is higher than in normal cells, a further increase in ROS and/or a decrease in antioxidant proteins may be potential strategies for glioblastoma treatment [12].

One of the most critical antioxidant systems in cells is the thioredoxin system [7], which includes thioredoxin (Trx) and thioredoxin reductase (TrxR). Trx is a peptide containing three SH groups that are sensitive to oxidation. Mammalian TrxR, a selenium-containing enzyme, catalyzes the reduction of oxidized Trx. TrxR exists in three isoforms: TrxR1 in the cytoplasm and extracellular space, TrxR2 in mitochondria, and TrxR3 in the testes [13]. While this enzyme participates in numerous cellular processes, including cell growth and differentiation [7], its primary function is to protect cells from oxidative stress. Furthermore, TrxR is highly expressed in mammalian brain cells [14].

Another important antioxidant system is the glutathione system, which consists of more components than the thioredoxin system does. Glutathione reductase (GR), the functional analog of TrxR, catalyzes the reduction of oxidized glutathione. Glutathione (GSH) is a water-soluble tripeptide composed of glutamine, cysteine, and glycine. The thiol group of cysteine can act as a reducing agent in redox reactions, making GSH the most abundant intracellular thiol, with concentrations reaching several millimoles in some tissues. GSH is synthesized de novo in cells through the sequential actions of two enzymes: γ-glutamylcysteine synthetase and glutathione synthetase [7]. Another component of the glutathione system is glutaredoxin. Unlike Trx, which is reduced by TrxR, glutaredoxin is nonenzymatically reduced via glutathione oxidation, with glutathione subsequently regenerated by GR [8].

Both antioxidant systems use electrons provided by NADPH, transferring them to glutaredoxins and terminal peroxidases, such as peroxiredoxins and glutathione peroxidases, which reduce ROS to nontoxic compounds [7,15]. Numerous studies have investigated the characteristics of these antioxidant systems in tumor cells, documenting the roles of both the glutathione [16,17,18] and thioredoxin systems [19,20,21] in conferring chemotherapy resistance. It was shown that increased activity of GSH related enzymes protects cancer cells and helps them to adjust to chemotherapy. In particular, it has been demonstrated that high intracellular GSH concentration in glioma cells is associated with chemoresistance and its depletion substantially enhances the cytotoxicity of cisplatin and TMZ [22,23]. Thioredoxin system is also important for supporting chemoresistance in gliomas. For patients with high-grade gliomas, both high cytoplasmic TrxR and Trx expression are associated with poor overall survival [24]. TrxR inhibition has been shown to sensitize glioma cells to chemotherapy leading to the development of novel treatments. Recent studies have demonstrated that TrxR inhibition by novel small molecules inhibitors can enhance the effectiveness of chemotherapy [25,26].

Affecting both antioxidant systems involves the transcription factor nuclear factor erythroid 2-related 2 (NRF2) pathway which is well known as the master regulator of antioxidant response, maintaining redox homeostasis in the cells [27,28]. NRF2 controls the expression of components of glutathione and thioredoxin systems [29]. NRF2 expression has a robust negative correlation with glioblastoma patient survival rates [30]. It limits the extent of autophagy induced by TMZ and protects tumor cells from the death induced by the drug [31]. The NRF2 induces GSH synthesis as a protective mechanism during TMZ treatment, silencing NRF2 potentiated TMZ-induced cell death [32]. Genome-Wide CRISPR screen has revealed that the NRF2 pathway contributes to TMZ resistance [30]. NRF2 has also been reported to be important for maintaining the stemness of glioblastoma stem cells (GSCs), which are one of the factors that contribute to GBM resistance to therapy [33].

The main challenge of GBM treatment is the heterogeneity of cell populations within this tumor type, which requires a multidrug approach. Recent studies in new drug development have mainly focused on targeting GSCs or cells in the tumor microenvironment, such as tumor-associated macrophages (TAM), T cells, or cancer-associated fibroblasts (CAF).

GSCs is a is a unique cell population within the tumor that were first identified as a subset of cells capable of initiating tumor growth in vivo [34,35]. These cells exhibit high transcriptional plasticity, proliferation capacity, and self-renewal potential [36,37]. Moreover, compared with more differentiated tumor cells, GSCs are characterized by an enhanced ability to invade surrounding tissues and have greater resistance to radiation and chemotherapy [38,39]. Numerous mechanisms underlying the increased resistance of GSCs to therapy have been described, including their altered energy profile, which allows them to survive increased intracellular ROS concentrations [40]. GSCs, however, are better capable of maintaining lower levels of ROS than are more differentiated tumor cells, which helps preserve genome integrity and supports their self-renewal [41,42]. Consequently, standard doses of TMZ may fail to induce cytotoxic effects in GSCs, necessitating additional strategies targeting the antioxidant systems that regulate ROS levels in these cells. Moreover, GSCs exhibit greater transcriptional plasticity than other tumor cells, making them better equipped to adapt to oxidative stress. These properties make GSCs highly resistant to therapy and the primary drivers of tumor recurrence. Elevated activity of antioxidant systems has been shown in cancer stem cells. It has been demonstrated that TrxR inhibitor could effectively eradicate cancer stem cells and particularly GSC by increasing ROS [43]. So, to overcome the therapeutic resistance of GSC inhibition of key cellular antioxidant systems particularly thioredoxin system could be a promising strategy.

Another important factor in glioma chemo- and radioresistance and a potential target for developing effective therapeutic strategies is a tumor microenvironment (TME). TME plays a vital role in the survival of cancer cells and their response to therapy. TME niche is comprised of many cell types, including endothelial cells, neural cells, CAFs [44], and resident and circulating immune cells and forms an environment that promotes and supports tumorigenesis [45].

ROS have a major impact on the functioning of TME. Elevated ROS level in particular CAFs, TAMs and T cells [9], have profound effects on tumor biology and demonstrated significant immunosuppressive effects [46]. First, CAFs and TAMs themselves can contribute to the formation of a pro-oxidant environment. It has been shown that CAFs express the enzyme NOX4 and generate intracellular ROS which are also necessary for maintaining their phenotype. CAFs suppress immune response by specifically excluding CD8+ T-cells from tumors and NOX4 is necessary for this activity [47]. Also, CAFs can recruit monocytes to TME and promote their differentiation into immunosuppressive TAMs. TAM generates ROS through activation of NOX-2 and also suppress CD8+ T-cell proliferation [48]. Also, macrophages contribute to drug resistance and relapse after chemotherapy treatment by promoting tumor revascularization and activating anti-apoptotic programs in cancer cells [49]. TAM are divided into tumor-suppressive M1-like (classic activation of macrophages) and tumor-supportive M2-like (alternatively activated macrophages) polarized cells [50]. The data on the role of ROS in induction of M1-M2 macrophage phenotypes are controversial. There are some data that ROS can stimulate both activation statuses in tumor-associated macrophages [51]. Increased ROS level may participate in inducing the polarization of macrophages from the phenotype M1 to M2 except for its involvement in tumorigenesis, thus causing suppressive tumor immune microenvironment [52,53]. It has been shown that in glioblastoma elevated oxidative stress and SOD3 are associated with increased M2-like pro-tumoral macrophages [54].

Despite the differences in the functioning of the TME during oxidative stress, certain adaptive mechanisms of the TME cell populations are required to avoid excessive ROS production and cell death. It was shown that thioredoxin system can support and protect the NK and T cells from elevated ROS levels [55]. Additionally, GSH depletion has been successfully used to overcome the immunosuppressive function of regulatory T cells (T regs) [56]. Wang et al. also demonstrated that the survival benefit of TME cells is accompanied by elevated expression of Trx [57]. So, the modulation of the ROS level by inhibiting the antioxidant system in cancer could be beneficial for glioblastoma therapy. This approach could have an impact not only on the tumor cells, but also on GSCs and the TME cells. However, there are dose-dependent effects of ROS on tumor biology. While a primary increase in ROS levels can lead to cancer cell death, it can also stimulate tumor progression in GSC or immunosuppressive mechanisms in the TME cells. All aspects of the redox status of different glioma cell populations should be considered when designing a multidrug treatment approach.

## 2. Inhibition of TrxR in Cancer

The thioredoxin-dependent system, in particular Trx, is considered a potential target for the development of effective anticancer drugs. Many TrxR inhibitors have been discovered and developed thus far, some of which have shown pronounced antitumor effects. Because of its high reactivity, TrxR can be inhibited by many electrophilic compounds, such as nitrosoureas, flavonoids, and metal-containing compounds, which are used in clinical practice. According to previous studies, TrxR inhibitors have high potential for improving the treatment of oncological diseases. They can potentiate the action of other cytostatic agents and overcome drug resistance in tumors, making them highly effective in combination therapy.

Alkylating agents—nitrosoureas—are irreversible inhibitors of TrxR. The nitrosoureas carmustine, lomustine and fotemustine covalently deactivate thioredoxin reductase, glutathione reductase and ribonucleotide reductase by alkylating their thiolate active sites [58,59]. Nitrogen mustards (chlorambucil and melphalan), cyclophosphamide [60,61] and alkyl sulfonates (busulfan) efficiently inhibit TrxR but do not inhibit glutathione reductase [59].

Arsenic trioxide is another irreversible inhibitor of TrxR with a mechanism involving both the C-terminal and N-terminal redox active sites of the enzyme [62]. Arsenic trioxide is a first-line treatment for acute promyelocytic leukemia and is also effective for other types of leukemia [63]. Arsenic trioxide has been shown to have anticancer activity in gliomas [64], breast cancer [65], hepatocellular carcinoma [66], lung cancer [67] and pancreatic cancer [68]. Phase I and pharmacodynamic study of arsenic trioxide showed promising results for patients with newly diagnosed GBM. The median overall survival for all patients in this trial was 17.7 months vs. 12 months for patients without the use of concurrent and adjuvant temozolomide [69].

Motexafin gadolinium (MGd) is a member of a class of rationally designed porphyrin-like molecules called texaphyrins. MGd preferentially spreads to cancer cells in animal models and in human clinical trials [70,71]. MGd acts as a substrate of cytosolic TrxR, and MGd and NADPH react in the presence of TrxR, producing ROS, which generate oxidative stress in cells [72]. In phase III clinical trials, MGd significantly prolonged the interval to neurologic progression in non–small-cell lung cancer patients with brain metastases receiving prompt whole-brain radiation therapy [73].

The natural compounds that exhibit inhibitory activity toward TrxR1 can be grouped as follows: phenylpropanoids, polyphenols, quinones, terpenoids, and chromones [74]. Curcumine—one of the most studied natural TrxR inhibitors causes alkylation of both residues in the catalytically active site (Cys(496)/Sec(497)) of TrxR [75]. It induces ROS production and elevates oxidative stress in cells [76]. These compound have an antitumor effect on xenografts in vivo [77].

Piperlongumine is a compound derived from long pepper plants. Piperlongumine inhibits cell growth and induces ROS accumulation in hepatocellular carcinoma cells. It induces a lethal endoplasmic reticulum stress response in HCC cells by targeting TrxR1 and increasing intracellular ROS levels [78].

Platinum compounds are potent TrxR inhibitors. They induce covalently stable intracellular TrxR1–Trx1 and TrxR1–TRP14 complexes [79]. TrxR, but not GR, is efficiently inhibited by cisplatin, carboplatin and oxaliplatin [59,80]. However, platinum-based compounds are only effective in a limited number of cancer types and have severe adverse effects [81]. Therefore, other metallodrugs, such as gold, mercury, copper and silver have been extensively studied.

Mercury (Hg) compounds such as thimerosal are rather quite effective because of their high affinity for binding to thiols and selenols. TrxR is highly sensitive to Hg inhibition because of the reactivity and position of the Sec residue in the open C-terminus of the TrxR active site [82,83].

Numerous gold (I) derivatives have been considered TrxR inhibitors including Au(I) complexes with phosphines (auranofin, triphenyl phosphine gold chloride, among others [84,85,86]), phospholes [87], thiomalate [84], thiosulfate [84], N-heterocyclic carbenes [88,89,90], azolates and phosphanes [91,92].

Gold (I) complexes sometimes also inhibit glutathione reductase [87] and show nonspecific reactions with thiols [93,94], which leads to cytotoxicity to normal cells.

Au(I) N-heterocyclic carbene (NHC) complexes are more cytotoxic to cancer cells than to normal cells and do not inhibit glutathione reductase [95]. A series of caffeine-based gold(I) NHC complexes have been synthesized and tested for their antiproliferative activities in different cancerous and nontumorigenic cell lines in vitro. The bis-carbene complex [Au(caffein-2-ylidene)2][BF4] appeared to be selective for human ovarian cancer cell lines and poorly toxic in healthy organs [88].

A bis-chelated tetrahedral gold(I) phosphine complex [Au(d2pype)2]Cl (where d2pype is 1,2-bis(di-2-pyridylphosphino)ethane) was designed to improve the gold(I) compound selectivity toward selenol- and thiol-containing proteins, such as TrxR. This compound is stable in the presence of serum proteins, thiols, or disulfides and exhibits high stability in solution [96]. The related bis-chelated Au(I) bidentate phosphine complex, 1,3-bis(di-2-pyridylphosphino)propane (d2pypp), also has unusually low thiol reactivity. It is selectively toxic to breast cancer cells but not normal breast cells, resulting in apoptotic programmed cell death [Au(d2pypp)2]Cl inhibits Trx and TrxR activity more in cancer cells than in normal cells. This difference appears to be in part a consequence of the increased uptake of the complex into cancer cells, particularly into their mitochondria [85]. It has been shown to exhibit anticancer effects against lymphoma [97], multiple myeloma [98] and chronic myeloid leukemia [99].

A series of water-soluble gold(I) compounds have been developed. Gold (I) complexes bearing water-soluble phosphine ligands, including 1,3,5-triaza-7-phosphaadamantane (PTA), 3,7-diacetyl-1,3,7-triaza-5-phosphabicyclo[3.3.1]nonane (DAPTA), and sodium triphenylphosphine trisulfonate (TPPTS), efficiently inhibited TrxRs at concentrations that did not affect glutathione reductase. These compounds are cytotoxic against cancer cell lines, particularly the cisplatin-resistant cell line [100]. Azolate gold (I) phosphane compounds have deactivating groups on the azole rings, which increases the solubility of these complexes in aqueous solutions. This gold (I) compound also has high cytotoxic activity for several human cancer cell lines in the low micromolar range and has significant selectivity for TrxR inhibition over GR inhibition [91]. Among other anti-TrxR inhibitors, AF plays a pivotal role in anticancer studies. It selectively inhibits Trx and TrxR and disrupts redox homeostasis leading to apoptosis in cancer cells.

## 3. The Anticancer Activity of AF

Numerous studies have demonstrated the anti-inflammatory, immunomodulatory, and antibacterial effects of AF. However, several studies have also highlighted the antitumor effects of AF in various models, both as monotherapy and in combination with other known antitumor agents. A few examples are presented in Table 1. Research papers have explored AF for the treatment of several types of blood cancer cells, including multiple myeloma [101], chronic lymphocytic leukemia [102], and chronic myeloid leukemia [103]. There is a constant search for new approaches to treating breast cancer (BC) due to its prevalence and rapid development of tumor resistance, which is characteristic of triple-negative BC (in which there is an absence of estrogen receptor, progesterone receptor, and receptor tyrosine-protein kinase gene expression). These tumors are not amenable to hormonal therapy, making it essential to suppress the development of drug resistance by identifying novel therapeutic strategies. One promising approach involves the use of a combination of antitumor drugs or mechanisms of action. For example, the combination of AF with antibodies targeting programmed death-ligand 1 (PD-L1) has been shown to significantly slow tumor growth [104]. For colon cancer therapy, AF (0.5 μM) was combined with 5Z-7-oxozeaenol (5 μM), a molecule capable of inhibiting transforming growth factor β-activated kinase 1 (TAK1). This combination led to cell death in colon carcinoma cells both in vitro and in vivo [105]. The activity of AF has also been reported in other cancer types, both in combination with known drugs and as monotherapy [106]. Of course, the most impressive results are from in vivo studies. Evaluating a series of data on the results of therapy in various tumor models, the following trends can be noted: the most effective therapy is therapy at doses ranging from 5 to 15 mg/kg with daily administration for 2 weeks. These methods make it possible to significantly reduce tumor growth and increase the survival of animals.

No more than 30% of the papers are devoted to GBM therapy by AF in Table 1. However, AF remains a promising drug for glioma therapy, which is confirmed by a significant number of in vitro studies on a variety of cell lines. For example, Ferraz et al. examined in detail the effect of gold and platinum complexes, where AF demonstrated one of the best abilities to inhibit TrxR and has a high cytotoxicity effect on the cells (U87, T-98, MRC-5) [127]. In another work, it was shown that the pro-oxidant Trx and/or GSH systems in U87MG and T98G cells were inhibited by AF, which caused increasing ROS, activating p53 and cell death [128]. The same results were demonstrated by Szeliga et al. on the U87MG, LN229, U87MG, LUB17, and LUB20 cells [129] and by Martinez-Jaramillo on the U87MG [130].

Notably, AF is being studied separately as an antitumor therapeutic agent and is currently in clinical trials. There are a number of clinical trials where AF was used as a potential drug for ovarian cancer (NCT03456700, NCT01747798), chronic lymphocytic leukemia (NCT01419691), non-small cell lung cancer (NCT02126527, NCT01737502), but the results of the clinical trials have not been posted. Furthermore, AF is being tested in combination with TMZ for the treatment of recurrent glioblastoma. This treatment protocol is called CUSP9v3, and in addition to AF, it includes eight other repurposed drugs in combination with TMZ. According to the CUSP9v3 protocol, after enrollment, the participant goes into the induction cycle, which lasts 35 days. The induction cycle consists of a drug-by-drug addition and up-dosing process. Hereafter, the subject will enter the treatment cycles (up to 12). During the induction cycle and the first 2 treatment cycles, regimen adjustments (dropping of certain drugs, dose modification of certain drugs) may be executed to accommodate to the patients’ individual toxicity reactions that may occur during this period. At present, the detailed results of the clinical trial (NCT02770378) of the CUSP9v3 protocol have not been posted. Although it is not the highest anti-cancer efficacy of AF, it has been shown to be a safe drug. In addition, AF can inhibit the Trx system in GBM cells, which makes AF a promising drug for GBM therapy in the near future.

## 4. AF Inhibits Thioredoxin Reductase Followed by the Activation of Other Pathways

**Figure 1:**
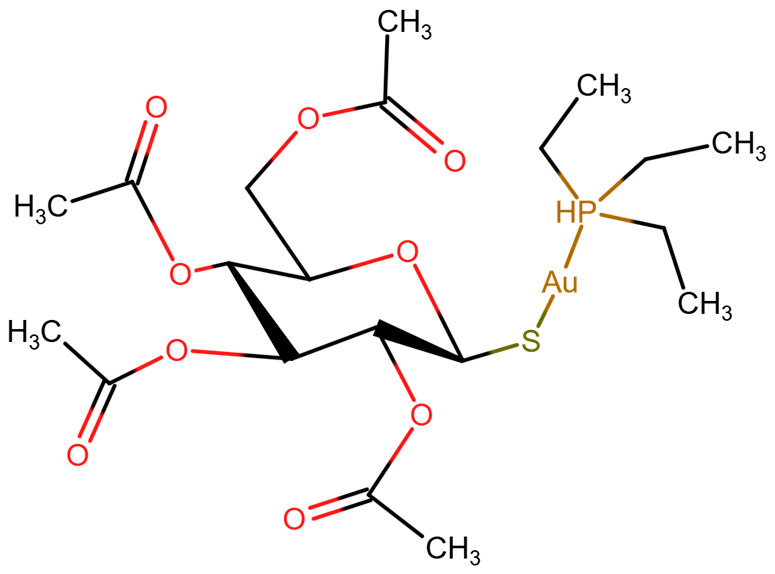
Chemical structure of auranofin.

AF (Figure 1), which first demonstrated clinical efficacy in 1976 [131], belongs to the group of gold-containing disease-modifying antirheumatic drugs.

The direct inhibition of TrxR by AF was first demonstrated by Stefan Gromer (Heidelberg University, Germany) and coauthors in 1998, and this mechanism is now widely accepted [132]. TrxR reduces Trx, a 12-kDa protein with a disulfide bond as its core functional group. The reduced form of Trx [Trx-(SH)2] restores oxidized protein substrates, which typically contain disulfide groups. The oxidized Trx form (Trx-[SS]) is regenerated by TrxR in an NADPH-dependent manner [133,134].

The thioredoxin reductase system, which includes Trx and TrxR plays a role in numerous biological processes, including redox homeostasis, antioxidant defense, transcription factor regulation, and cellular proliferation and division [135]. The inhibition of TrxR1, which leads to increased ROS levels, is currently recognized as the primary mechanism of AF cytotoxicity [106]. In vitro studies have also shown that AF inhibits other thioredoxin reductases, such as TrxR2 and TrxR3 [136,137].

Researchers have discovered that TrxR provides protective effects against various cellular stresses, including growth inhibition and cell death induced by hydrogen peroxide, tumor necrosis factor-α, and chemotherapeutic agents [138,139,140]. The literature indicates that not all cancer tumors exhibit the same level of TrxR expression, affecting their sensitivity to AF. For example, cisplatin-resistant human bladder cancer cells and PC-3 prostate cancer cells are characterized by elevated TrxR expression levels [138,141,142].

Conversely, tumor cells with high ROS levels may be more vulnerable to cell death when antioxidant systems are inhibited [143]. Therefore, a potential cancer treatment strategy involves selectively disrupting the redox balance of pathological cells by suppressing their antioxidant systems while sparing healthy cells [144].

Research, including bioinformatics approaches, is ongoing to identify alternative mechanisms of action of AF. For example, researchers from Peking University have shown in vitro and in vivo that AF inhibits the catalytic activity of TET1 (ten-eleven translocation 1). TET1 is a key epigenetic regulator of DNA that catalyzes the conversion of 5-methylcytosine (5mC) to 5-hydroxymethylcytosine (5hmC) to modulate gene expression. Analysis of public datasets (GSE5820) revealed that TET1 expression is elevated in patients with T-cell acute lymphoblastic leukemia (T-ALL) and serves as a biomarker for decreased survival. The competitive binding of AF to the substrate pocket of TET1 reduces 5hmC levels, leading to epigenetic reprogramming of the oncogene c-Myc. Thus, AF was shown to inhibit TET1 in T-ALL models via the TET1/5hmC/c-Myc signaling pathway [145].

Using a large transcriptomic dataset (9 cell lines: A375, A549, HCC515, HEPG2, HT29, MCF7, PC3, HA1E, and VCAP before and after AF exposure) and Clue software (clue.io/cmap, [146]), 978 key genes were identified that characterize the inhibitory effects on TrxR. Gene expression changes were compared to all available gene signatures in the database (over 1 million profiles), generating a “tau ball” that predicted the effect.

NF-κB inhibition showed the greatest predictive power. In 2000, Jeon et al. demonstrated that NF-κB is inhibited by 5–10 μM AF over 4 h in macrophages stimulated with lipopolysaccharide, blocking IκB kinase activity [147]. Nakaya et al. reported that 0.05 μM AF inhibits NF-κB DNA binding and reduces nuclear NF-κB levels in U266 multiple myeloma cells [101]. AF is thought to bind to the Cys179 residue of IκB kinase, blocking its activity. IκB kinase phosphorylates IκB, which binds to NF-κB, causing dissociation of the IκB + NF-κB complex. Once dissociated, phosphorylated IκB detaches from NF-κB, which migrates to the nucleus to activate transcription [148].

Interestingly, IκB is not the only substrate of IκB kinase. Hu et al. reported that it also phosphorylates the tumor suppressor FOXO3, which becomes inactive upon phosphorylation and translocates to the cytosol [149]. Thus, AF-induced IκB kinase inhibition may produce various responses, with cytotoxicity potentially dependent on specific cell types and tumor microenvironments in vivo.

Another proposed mechanism based on bioinformatics analysis involves the inhibition of deubiquitinating enzymes (DUBs), which remove ubiquitin degradation tags from proteins, preventing their proteasomal degradation. AF inhibits DUBs (UCHL5 and USP14) in the 16S proteasome regulatory subunit at 0.5 μM over 3 h, as demonstrated in HEPG2 and MCF7 cells [150]. These findings were later confirmed in prostate cancer models in vitro and in vivo [151]. However, recent proteomic research suggests that AF-dependent DUB and proteasome inhibition occur only at high doses (>2.5 μM) and may represent off-target effects [152].

In 2013, Wang et al. proposed a direct interaction between AF and protein kinase C-iota (PKCiota), as AF analogs (aurothiomalate and aurothioglucose) inhibit this enzyme by binding critical cysteine residues [153,154]. PKCiota mRNA levels are elevated in various cancers, but the inhibitory effect of AF on this kinase requires further study [155].

Hou et al. demonstrated that AF inhibits hexokinase at 4–6 μM, reducing glucose uptake, lactate production, and ATP synthesis in vivo [112]. Since hexokinase is a key glycolytic enzyme that is overexpressed in many cancers, its inhibition could decrease ATP production and impair the ABCG2 pump, which contributes to multidrug resistance in chemotherapy [156]. The targeting of hexokinase is considered a promising anticancer strategy, but further data on the role of AF in this process are needed.

**Figure 2:**
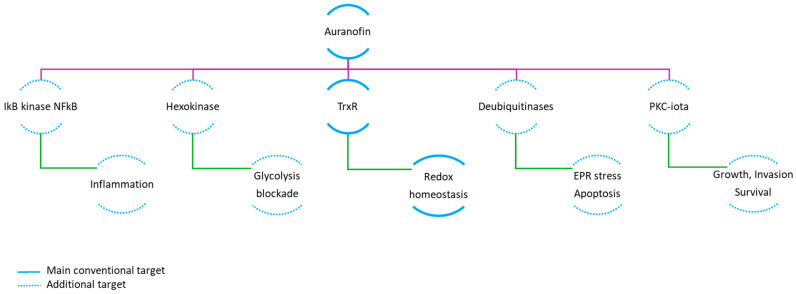
Summary of the main molecular targets affected by auranofin. Abbreviations: NFkB—Nuclear factor kappa-light-chain-enhancer of activated B cells; PKC-iota—Protein kinase C iota; TrxR—thioredoxin reductase; EPR—endoplasmic reticulum. Solid line stands for the conventional mechanism, dotted for additional.

In conclusion, on the basis of research conducted in various cancer models, several mechanisms, including dose-dependent effects, have been proposed to explain the antitumor activity of AF. The main direct and indirect targets of AF are summarized in Figure 2 [106].

## 5. Formulation and Effective Delivery of TrxR Inhibitors (AF) for Brain Tumors

The development of new drug forms for glioma treatment remains a complex challenge. There are three main issues associated with new drugs: stability, solubility and delivery.

An AF, an inhibitor of TrxR, is soluble in organic solvents such as ethanol and DMSO but insoluble in aqueous solutions, which can hinder its bioavailability in vivo and lead to complex formulation protocols [100]. The formulation of AFs is critical for treating brain pathologies, as it has another drawback: it reacts nonspecifically with protein thiols [93,94], which can make it toxic to normal cells. To address this issue, several approaches can be employed, such as increasing solubility by solubilizing agents or encapsulating the active pharmaceutical ingredient (API) in nanoparticles. Encapsulating AF into nanoparticles or combining it with other pharmaceutical excipients can minimize its potential adverse effects, preserve its interaction with serum proteins, and result in greater stability. This approach reduces the amount of free drug circulating in the body, thereby preventing unwanted tissue delivery and improving therapeutic efficacy [157].

The first approach (using solubilizing agents) involves the use of different expedients to improve the stability and solubility of AF. Typical excipients for improving the solubility of poorly soluble drugs for oral and intravenous administration include the following classes of compounds [158]: (1) water-soluble organic solvents (PEG 300 or 400, ethanol, propylene glycol, glycerol, N-methyl-2-pyrrolidone, dimethylacetamide and DMSO); (2) nonionic surfactants (Cremophor EL or RH 40 or RH 60, polysorbate 20 or 80, poloxamer 407 and mono- and differential acid esters of PEG); (3) water-insoluble lipids (castor oil, corn oil, olive oil, etc.); (4) organic liquids/semicolids (beeswax, d-α-tocopherol, oleic acid, medium-chain mono- and diglycerides), and (5) various cyclodextrins (β-cyclodextrin, α-cyclodextrin, hydroxypropyl-β-cyclodextrin). However, at present, few studies have described drug forms based on AF via the abovementioned substances for i.v. injection.

Nanoparticles are another form of excipients that should protect the API from degradation, ensure controlled release, enhance solubility, and reduce overall toxicity. Over the past 20 years, various drug delivery methods, including gels, micelles, polymer particles, PLGA particles, and inorganic particles (e.g., transition metal oxides or noble metals), have been proposed. These approaches enable the delivery of small molecules such as cytostatic or larger entities such as proteins, antibodies, RNA, or viruses. Owing to their relatively simple synthesis and availability of clinical trial data, liposomes and polymer nanoparticles are the most common carriers. Other promising delivery systems include gold, magnetic, and mesoporous silica nanoparticles. Magnetic and gold nanoparticles rely on coatings for controlled drug release, whereas mesoporous silica nanoparticles utilize their nanopores for controlled release. The addition of vector molecules and surface chemistry modifications further enhances penetration and therapeutic outcomes, as demonstrated in studies using magnetic nanoparticles loaded with TMZ [159]. However, the most popular method for brain delivery is the encapsulation of API into cationic liposomes [160].

There are several examples of formulations of AF using nanoparticles. One interesting example of nanoparticle use involves chitosan-PEG copolymers. Maame Abena O. Afrifa et al. demonstrated improved solubility and greater efficacy at lower doses of AF (3 mg/kg vs. 5 mg/kg) for the treatment of triple-negative breast cancer [161]. Another study by Marta Pérez-Lloret showed that silk fibroin nanoparticles loaded with AF improved its bioavailability, cellular absorption, and resistance to degradation, successfully inhibiting the growth of 3D colorectal carcinoma cultures [162]. AF also exhibit antimicrobial activity. Encapsulating it in PLGA nanoparticles enhanced its bactericidal effects, virtually sterilizing multiresistant pneumococcal strains (e.g., *Streptococcus pneumoniae* and *Streptococcus pyogenes*) after six hours of treatment at 0.25 μM. This potent effect was also observed in biofilms, where AF-NPs reduced bacterial populations by approximately four logarithms more than free AF did [163]. Interestingly, PLGA nanoparticles loaded with AF have also been used as neuroprotective agents for Alzheimer’s disease treatment. These nanoparticles significantly reversed cognitive deficits, biochemical changes, neuroinflammatory markers, and neurotransmitter imbalances in a streptozotocin-induced model [164]. Notably, similar particles loaded with other drugs have already been delivered to the brain, which points to the possibility of delivering AF to the brain tumors in the form of a nanoparticle formulation. However, several approaches can improve the delivery of nanoparticles.

Although AF and AF-loaded nanoparticles have shown significant efficacy in preclinical studies on non-brain tumors, there are still limited data regarding their effectiveness in GBM studies. AF has already been shown to be effective as a treatment for rheumatoid arthritis, but the anticancer activity of AF requires higher doses. A major challenge in developing new drugs for GBM is achieving sufficient therapeutic concentrations in the brain, which is often hindered by the low solubility of the drugs and the limited BBB permeability. To achieve the maximum concentration, several approaches could be used: (1) the addition of lipid-like molecules through modification of the hydrophilic groups on the drug structure [165]; (2) the addition of hydrophobic groups to the drug molecule [166]; (3) active targeting; (4) opening of the BBB (FUS, osmotic opening); and (5) intraarterial/intrathecal administration.

In the first case, drug permeability across the BBB is favored by their lipophilicity, low molecular weight, and lack of ionization at physiological pH. Lipid-soluble molecules with a molecular weight of <500 Da may cross the BBB through the small pores that form transiently within the lipid bilayer [165]. An example of this type of modification is the addition of methyl groups to various drugs in the barbiturate class, which results in increased lipophilicity and brain penetration in animals [166]. A more recent approach is to enclose already known API in liposomes (mostly cationic). For example, improved drug delivery has been demonstrated for encapsulated doxorubicin in the treatment of experimental brain tumors [167].

The use of targeting moieties such as transferrin, insulin, apolipoproteins, and antibodies attached to polymeric nanoparticles for maximum accumulation has been demonstrated [168]. For example, transferrin strongly influences the binding of magnetic PLGA particles to glioma cells, and the results of in vivo experiments revealed that particles with transferrin loaded with doxorubicin and paclitaxel significantly suppress tumor growth [169]. Increased permeability and improved therapeutic efficacy due to the presence of transferrin on the nanoparticles have also been reported in other studies. Thus, the efficacy of zoledronic acid in the treatment of GBM was increased by encapsulating it in self-assembling nanoparticles with transferrin, which allowed the drug to have improved solubility and imparted the ability to penetrate the BBB [170].

While active targeting could enhance the accumulation of drugs in brain pathology, it also presents some challenges (difficulties in manufacturing and scaling). Other methods to increase the therapeutic concentration of a drug in brain tissue include temporary BBB opening via clinically approved focused ultrasound (FUS) [171] or osmotic opening (mannitol) [172]. Temporal BBB opening after exposure to ultrasound is possible after the injection of gas-filled microbubbles (e.g., sulfur hexafluoride). The use of microbubbles safely opens the BBB [173] and the absence or minimal damage to nervous tissue [174]. To date, more than 10 microbubbles have been registered for clinical practice, and several clinical trials have focused on the efficacy of drug therapy via focused ultrasound to improve the penetration of drugs (TMZ, doxorubicin, carboplatin, etc.) into brain tumors [175]. Additionally, this approach has been shown to be effective for the delivery of nanoparticles in preclinical trials. Thus, exposure to ultrasound leads to a 6–40-fold increase in the accumulation of particles with cisplatin (60-nm particles) [176], a 1.4–6.9-fold increase in the accumulation of particles with paclitaxel (170-nm particles) [177], and a 2-fold increase in the accumulation of nanoparticles with carmustine (10–20-nm particles).

In conclusion, there are various approaches thar could improve the efficacy of AF in clinical application. There are numerous methods for enhancing the delivery of AF across the BBB for the treatment of GBM. These methods can be categorized into two types: first, improving the formulation of the substance itself, either by adding specific functional groups or encapsulating it in various novel drug forms; second, temporarily increasing the permeability of the BBB through artificial means. Also, the antitumor efficacy of AF could be improved by modulation of dynamic thiol exchange of gold ions, which leads to a fast formation of covalent albumin-gold adducts. Researchers from Sun Yat-Sen University report that AF supplemented with its own thiol ligand, TGTA (1-thio-β-D-glucose tetraacetate) significantly restored anticancer activity in cells and patient-derived xenograft models [178]. Screening a library of ligand fragments and conducting machine learning analysis afterward may be a promising strategy for enhancing the effectiveness of AF even at a reduced dosage. In addition, using AF to sensitize cancer cells to other approved drugs or in combination with other anti-cancer therapies could be a promising approach. Combination therapy with AF demonstrated good results in therapy, including GBM [109,110]. Taken together, these approaches provide hope for the potential success of GBM treatment via AF, as AF has excellent potential to inhibit TrxR.

## 6. Conclusions

GBM is a challenging disease that has various cell populations with different redox statuses. GSCs and TME cells have become attractive targets for developing anticancer therapies while playing a key role in tumor relapse. These cell populations benefit from elevated ROS production and develop adaptive mechanisms to survive through high ROS level, such as strong antioxidant systems. One approach to targeting cancer cells is by suppressing their antioxidant system in the cell by inhibiting TrxR. AF has shown promising results as a selective inhibitor of TrxR, demonstrating anticancer activity against various tumor types, including GBM. However, there are some challenges in further clinical application for GBM that should be overcome, such as the low solubility of AF and poor delivery. While its solubility in water is limited, studies have explored the development of new formulations of AF suitable for intravenous administration. These formulations could potentially allow AF to cross the blood-brain barrier and be used in the treatment of GBM in the future.

## Figures and Tables

**Table 1:** Potent anticancer activity of AF in preclinical tumor models. Abbreviations: i.p. (intraperitoneal injection), i.v. (intravenous injection), p.o. (per os, oral administration).

Compound	Solvent (for AF)	Model	Cells	Treatment Protocol (Groups and Doses)	Effect of Tumor Growth Inhibition	Mechanism	Ref.
AF	(1) 4% (*v*/*v*) DMSO/10% (*v*/*v*) ethanol (2) 50% (*v*/*v*) DMSO, 40% (*v*/*v*) PEG300 and 10% (*v*/*v*) absolute ethanol	C57BL/6J and 129S2/SvPasCrl	SB28 and 344SQ	Doses: 2, 5, 10 and 15 mg/kg (1) i.p. injections over a period of 14 consecutive days; (2) s.c. via osmotic minipumps for 14 days; (3) p.o. for a period of 14 days	Daily i.p. injections of 10 mg/kg AF induced weight loss and gastro-intestinal problems. High doses of AF were able to inhibit TrxR activity in SB28 tumors after 14 days. A solvent consisting of 50% DMSO, 40% PEG300 and 10% ethanol provided optimal solubility of AF for p.o. administration to mice	inhibited TrxR activity	[107]
AF	Non-metioned	Athymic nude mice (Foxn1nu)	LN229, U251 and P3	AF (5 or 10 mg/kg/day) or PBS, p.o. starting at day 7 after implantation	AF inhibited GBM progression in vivo	Inhibited SC35 agglomerates, prevented NONO; binding to pre-mRNA and promoted the degradation of NONO protein	[108]
AF	DMSO and corn oil (1:4 ratio).	BALB/c nu/nu mice	TGS-01	Nigericin (4 mg/kg/day, i.p. injection, every 2 days) or AF (12 mg/kg/day for 2 days, i.p. injection)	AF inhibited GBM progression in vivo	Decreased ATP levels, induced abnormality in mitochondrial membrane potential	[109]
AF and AF with saracatinib	DMSO	Pten; Trp53; NOD-SCID	Human GBM	12 mg/kg by i.p. injection, 6 days per week and or AF + saracatinib (12 mg/kg by i.p. injection 6 days/week; 17.5 mg/kg by p.o. 5 days/week, respectively)	Increased median survival compared to the placebo group	Inhibition PKCι	[110]
AF	5% DMSO, 10% Cremophor EL, 12.5% PEG, and 15% Ethyl Alcohol, and 57.5% H_2_O	BALB/c nude mice	MiaPaCa-2	5–15 mg/kg AF, i.p. injection, 5 times per week for 21 days	15 mg/kg is the optimal dose: median survival increased to 12 days (versus 8 days in control); suppression of gross abdominal metastasis and a low occurrence of ascites	TrxR1 inhibition	[111]
AF and adriamycin (ADM)	DMSO	BALB/c nude mice	A549	(1) 10 mg/kg AF, i.p. injection, five times per week for 6 weeks, (2) ADM 5 mg/kg, i.v. injection, once per week for 6 weeks. (3) combination of AF and ADM	treatment with AF or ADM alone exhibited a moderate inhibitory effect on tumor growth. Combination of both drugs resulted in a significant decrease in tumor growth.	ROS increase, consistent with inhibition of TrxRs, inhibition of glycolytic hexokinase	[112]
AF and anti-PD-1 antibody	DMSO	BALB/c nude mice (human JeKo-1) and immunocompetent Balb/c (A20)	human JeKo-1 and A20	Jeko-1 model: 10 mg/kg AF, i.p. injection, 5 times per week. A20 model: (1) control, (2) anti-PD-1 antibody, (100 μg/mouse, i.p. every 5 days); (3) AF (10 mg/kg/day, i.p.); (4) combination of AF and anti-PD1 antibody	AF and anti-PD-1 antibody induced significant apoptosis in lymphoma tissues	Elevated ROS prouction, inhibiting ATP generation and induction of PD-L1 expression	[113]
AF	DMSO	BALB/c nude mice	NCI-H460	10 mg/kg AF, i.p. injection, every 2 days for 14 days	Tumor growth inhibition; AF increased the expression of apoptosis-related proteins	TrxR1 expression inhibition	[114]
AF	DMSO	BALB/c nude mice	PSN1	12.5 mg/kg AF, i.p. injection, 5 times weekly for 5 weeks	AF suppressed tumor growth of ~40% of vehicle control	Inhibited TrxR activity	[115]
AF and piperlongumine (PL)	DMSO	BALB/c nude mice	SGC-7901	i.p. injection once per day, groups: (1) 4 mg/kg PL, (2) 2 mg/kg AF (3) combination AF and PL (total 14 days)	combined treatment with AF and PL significantly inhibited tumor volume and tumor weight	Inhibited TrxR activity	[116]
AF and cold atmospheric plasma (CAT)	DMSO	C57BL/6J mice	SB28 glioma	(1) 15 mg/kg AF, p.o., daily for 14 days (2) CAT for 5 days (3) combination of AF and CAT	the sequential combination regimen resulted in a significantly decreased tumor volume and significantly increased survival of the SB28-bearing mice	Inhibited TrxR activity	[117]
AF and celecoxib (CE)	olive oil	BALB/c nude mice	DLD-1	Six groups of mice were treated as follows (p.o., 30 days): (1) solvent control (olive oil); (2) AF 10 mg/kg; (3) CE 20 mg/kg; (4) CE 60 mg/kg; (5) AF 10 mg/kg + CE 20 mg/kg; (6) AF 10 mg/kg + CE 60 mg/kg.	The combination of AF and CE showed significant inhibition of tumor growth	Inhibition of hexokinase enzyme activity; increase in ROS accumulation	[118]
AF, carboplatin and buthionine-sulfoximine (BSO)	ethanol and Cremaphor EL	BALB/c nude mice	A549; H292	(1) 450 mg/kg BSO (2) 1.6 mg/kg AF (3) combination of AF and BSO i.p. daily for two weeks. (4) combination AF, BSO and carboplatin 5 days a week for 2 weeks.	Au+BSO+carbo resulted in a highly significant decrease in tumor growth rate when compared to control, carboplatin or combination of AF+BSO	inhibited TrxR activity	[119]
AF and cisplatin	DMSO	BALB/c nude mice	H69	Mice treated by i.p. injection every two days for 28 days with (1) DMSO as control, (2) AF (10 mg/kg), (3) cisplatin (2 mg/kg) and (4) combination of AF with cisplatin	The combination treatment with AF and cisplatin significantly inhibited tumor volume of H69 xenografts as compared with vehicle treatment. AF or cisplatin single treatment did not significantly inhibit tumor growth.	Enhanced cisplatin-induced S-phase cell cycle arrest in cisplatin-resistant H69 and H196 cells; increase of ROS accumulation	[120]
AF and ganetespib	20% Cremophor RH40 and 80% D5W i.v. injection and 0.5% hydroxypropyl methylcellulose in 5% dextrose	BALB/c nude mice	A673	(1) 12 mg/kg AF, i.p. injection, 5 days per week, (2) 150 mg/kg ganetespib, i.v. injection, and (3) combination of AF and ganetispib once weekly for up to 18 days	The survival rate in the combination group was nearly doubled in this extremely aggressive EWS tumor animal model compared to the control group	Inhibited TrxR activities	[121]
AF and 5Z-7-oxozeaenol (TAK1 inhibitor)	sterile 2.5% DMSO in vegetable oil	BALB/c nude mice	SW 620	(1) vehicle control (2) 1.6 mg/kg AF (3) 5Z-7-oxozeaenol 15 mg/kg, (4) combination of AF and 5Z-7-oxozeaenol, The drugs were i.p. injected for five days, followed by 2 days of AF only, and then four days of combination treatment.	Auranofin alone did not significantly slow tumor growth as compared to the control group. The combination of 5Z-7-oxozeaenol plus AF showed the slowest tumor growth, however this did not reach statistical significance as compared to TAK1 inhibitor alone	Inhibited TrxR activities	[105]
AF and 5-fluorouracil (5-FU)	DMSO	BALB/c nude mice	SW620/5-FU	(1) 5-FU: 50 mg/kg, once every 4 days, i.p.; (2) 6 mg/kg AF, daily, intragastric (3) combination of FU + AF for 24 days	Animals in the 5-FU+AF treatment group had fewer lung metastatic nodules than in the 5-FU or AF groups, less necrosis was observed in the drug combination group than in either of the single-drug groups.	Inhibited TrxR activities and FoxO3-promotion	[122]
AF and selenocystine (SeC)	DMSO	BALB/c nude mice	A549	(1) 5 mg/kg SeC (2) 2 mg/kg AF (3) combination of SeC and AF, i.v. injection, daily for 16 days	Combined treatment with SeC and AF significantly inhibited the tumor weight and tumor volume but did not affect the body weight of mice.	inhibited TrxR activities; induction of mitochondria-mediated apoptosis confirmed by caspases activities	[123]
AF	2% DMSO, 8.5% ethanol, and 5% PEG-400	BALB/c nude mice	Calu3	(1) AF (10 mg/kg/day) and (2) solvent, i.p. injection, daily for 14 days	Treatment with AF resulted in significant growth suppression of Calu3 tumors and led to 67% inhibition of tumor growth compared with control. No weight loss was detected.	PI3K/Akt/mTOR pathway and NRF2-mediated oxidative stress response inhibition	[124]
AF	DMSO (10%)	BALB/c mice	CT26	AF 10 mg/kg/day i.p. 3 times a week for 3 weeks	The treatment reduced tumor growth and preserved body weight in mice. Also, administration of auranofin before irradiation enhanced the radiation response of colon tumors while providing radioprotection to normal tissues, including the small intestine.	Activated the p53/p21 pathway	[125]
AF	DMSO (5 mmol/L)	TCL-1 transgenic mice (Tcl1-tg:p53−/−)	CLL (chronic lymphocytic leukemia) cells	AF 10 mg/kg i.p. daily, 5 times per week for 2 weeks	Treatment with AF resulted in a reduction in leukemia cell burden in every TCL-1 mouse tested and improved mouse survival	Inhibited TrxR activity	[126]

## Data Availability

Not applicable.
